# Effect of clonidine on blood glucose levels in euglycemic and alloxan-induced diabetic rats and its interaction with glibenclamide

**DOI:** 10.4103/0253-7613.58510

**Published:** 2009-10

**Authors:** S. Manjunath, Santosh N. Kugali, Priyadarshani M. Deodurg

**Affiliations:** Department of Pharmacology, M.R. Medical College, Sedam Road, Gulbarga - 585 105, India

**Keywords:** Clonidine, glibenclamide, hyperglycemia, hypoglycemia

## Abstract

**Objectives:**

Clonidine, a known antihypertensive, is currently used for many purposes including diabetic gastroparesis, postmenopausal hot flushes, opioid/nicotine/alcohol withdrawal. Its effects on carbohydrate metabolism appear to be variable. Hence, the present study was undertaken to evaluate the influence of clonidine on euglycemic and alloxan -induced diabetic rats and its interaction with glibenclamide.

**Materials and Methods:**

Alloxan - induced (150 mg/kg, i.p) diabetic rats were divided into six groups of six animals each. Group I - Normal Control; Group II - Nondiabetic + Clonidine (25 μg/kg); Group III - Diabetic Control; Group IV - Diabetic + Glibenclamide (5 mg/kg); Group V - Diabetic + Glibenclamide + Clonidine. All drugs were given orally once daily. Blood glucose was estimated from rat tail vein using glucometer before start of the experiment and at the end of 30 days.

**Results:**

After 30 days of treatment, clonidine (25 μg/kg) produced significant hyperglycemia in both euglycemic and diabetic rats. It also reduced the hypoglycemic effect of glibenclamide in diabetic rats.

**Conclusion:**

The results of present study indicate that clonidine has hyperglycemic effect and it also interacts with glibenclamide to reduce its hypoglycemic activity. If these findings are true to human beings then clonidine should not be used in diabetic patients on sulfonylureas.

## Introduction

Diabetes mellitus has emerged as a major public health problem in the developing world. It is associated with many co-morbid conditions such as hypertension, obesity, cardiovascular disorders, dyslipidemia and others. Sulfonylureas are commonly used as oral antidiabetic drugs in the treatment of Type 2 diabetes. Concomitantly administered drugs to treat associated conditions may influence the hypoglycemic action of oral antidiabetic drugs.

Today, with the advent of newer antihypertensive drugs, the use of clonidine in treatment of hypertension is limited. However, the list of newer uses of clonidine is increasing rapidly. Nowadays, clonidine is more commonly used in the treatment of diabetic gastroparesis, postmenopausal hot flushes, alcohol/nicotine/opioid withdrawal symptoms, in preoperative and postoperative anesthesia and analgesia.[1]

The effects of clonidine on carbohydrate metabolism appear to be variable. Clonidine inhibits insulin secretion from pancreatic beta cells possibly via α_2A_ receptor.[2,3] Some studies suggest that clonidine does not affect carbohydrate metabolism in diabetic[4] or non-diabetic hypertensive patients.[5] Conversely, studies have also shown that clonidine caused hyperglycemia in human beings and experimental animals.[6,7] In view of contradictory reports regarding the hyperglycemic activity of clonidine and paucity of information regarding its interaction with commonly used sulfonylurea drugs such as glibenclamide, the present study was taken up; to investigate the effect of clonidine on blood glucose levels in euglycemic and alloxan-induced diabetic rats and to know the interactions of clonidine with glibenclamide.

## Materials and Methods

The study was undertaken at the Department of Pharmacology, M. R. Medical College, Gulbarga, after obtaining the approval from Institutional Ethics Committee. Adult albino rats weighing approximately 200-250 g were used for the study. Diabetes was induced by a single dose (150 mg/kg, i.p) of freshly prepared solution of alloxan monohydrate 5% (dissolved in normal saline). The induction of diabetes was confirmed after 48 h by blood glucose estimation and rats with blood glucose levels between 250-350 mg/dl were selected for the study. The animals were divided into six groups of six animals each as below:

Group I: Non diabetic rats treated with normal saline.

Group II: Non diabetic rats treated with clonidine (Unichem Lab Ltd Mumbai) (25 μg/kg)[8]

Group III: Diabetic rats treated with normal saline

Group IV: Diabetic rats treated with glibenclamide (Hoechst India Ltd. Mumbai) (5 mg/kg)[9]

Group V: Diabetic rats treated with clonidine (25 μg/kg).

Group VI: Diabetic rats treated with glibenclamide (5 mg/kg) and clonidine (25 μg/kg).

All the animals were maintained under standard animal house conditions with free access to food, water and ad libitum. All the drugs were administered orally (using a polythene tubing sleeved on an 18-20 gauge blunted hypodermic needle) in a single dose in the morning.

Blood glucose was measured before starting the treatment and at the end of 30 days (end of the treatment). Due care was taken like housing only 3 rats per cage, using sterile instruments and changing the husk twice daily to reduce the risk of infections.

### Method of blood collection

Blood sample for glucose estimation was collected from rat tail tip. In a well restrained rat, the tail was embedded in hot water (45°C) and about 1 mm of its end was cut and the drop of blood was collected directly on the strip placed in the glucometer (One Touch Horizon, Life Scan Inc. Milpitas USA, Johnson and Johnson Company).

### Statistical analysis

The results obtained were analyzed using Student *t* test and *P* value less than 0.05 was considered significant.

## Results

**Figure 1:**
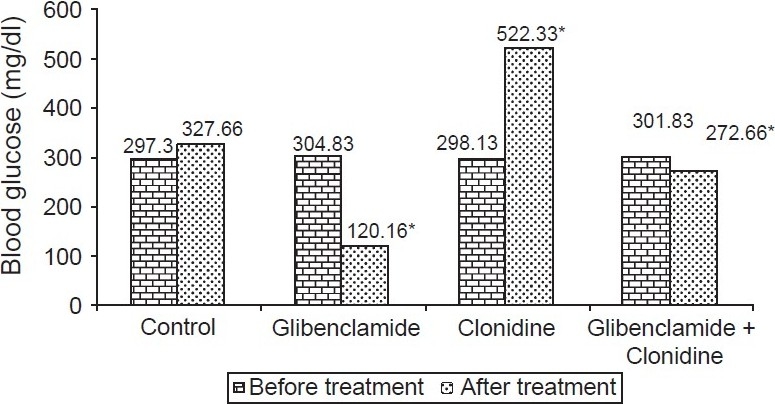
Effect of clonidine and glibenclamide on blood glucose levels in diabetic rats; n = 6; *P ≤ 0.001 as compared to control (Student *t* test)

The control group (Group I) had no change in blood glucose levels. Clonidine -treated non diabetic group (Group II) had a significant (*P* < 0.001) increase in the blood glucose levels and percentage increase was 108.31% as compared with the control group. The diabetic control (Group III) had an increased blood glucose levels. There was a significant (*P* < 0.001) decrease in blood glucose levels of diabetic group treated with glibenclamide (Group IV). The clonidine -treated diabetic group (Group V) showed significant (*P* < 0.001) rise in blood glucose levels. The glibenclamide plus clonidine treated group (Group VI) had a significant decrease in blood glucose levels (*P* < 0.001) [Figure 1].

## Discussion

Previous studies have shown that clonidine causes hyperglycemia in non -diabetic mice[10] and in normotensives. Clonidine is also known to be associated with elevated fasting blood glucose in a diabetic patient and increases insulin requirements.[11,12]

In contradiction to these reports, beneficial effects of clonidine on glycemic control such as prevention of insulin resistance and diabetes induced hyperglycemia have also been reported.[13,14] Meanwhile in our study, treatment with clonidine has significantly increased the blood glucose in alloxan -induced diabetic rats. Another study indicates beneficial interaction between clonidine and glibenclamide wherein, clonidine intensified fall in blood glucose levels of rabbits treated with glibenclamide.[15] In a study on diabetic hypertensive patients, clonidine did not appear to alter the diabetic control by oral hypoglycemic agents.[16] Thus, the reports are inconclusive and ambiguous.

The results of the present study differ from the above observations. In our study, significant hyperglycemia is evident in both euglycemic and diabetic rats treated with clonidine alone. While the group treated with a combination of clonidine and glibenclamide showed no beneficial interaction, instead clonidine reduced the hypoglycemic effect of glibenclamide. This antagonizing effect can be explained by the result which shows significant hypoglycemia (60.58% reduction) with glibenclamide alone whereas, after addition of clonidine with glibenclamide the hypoglycemia was less (9.5% reduction). Therefore, the present study proves the hyperglycemic effect of clonidine in both euglycemic and diabetic rats and its interaction with glibenclamide.

The hyperglycemia could be due to inhibition of insulin release, stimulation of growth hormone release and due to increased hepatic glycogenolysis by clonidine.[17,18] The interaction is probably pharmacodynamic in nature.

Although clonidine is not commonly used as an antihypertensive, recent studies have shown many utilities of clonidine in : Diabetic gastroparesis, peripheral neuropathy, migraine, opioid/alcohol/nicotine withdrawal, post menopausal hot flushes, ADHD, preoperative and postoperative anesthesia and analgesia, bipolar disorder etc.[1] Thus in any situation where clonidine is used in a diabetic patient who is on sulfonylurea, there is a possibility of interaction leading to the decreased effect of sulfonylurea.

## Conclusion

Clonidine causes rise in blood glucose levels in normal as well as alloxan -induced diabetic albino rats. It also reduces the hypoglycemic effect of glibenclamide in diabetic rats. If these findings are true to human beings, clonidine should not be used in diabetic patients on glibenclamide therapy as it may reduce the hypoglycemic effect of glibenclamide.

Further studies are required to understand the exact mechanism of this interaction and its clinical implications.
